# Integrating gene expression and splicing dynamics across dose-response oxidative modulators

**DOI:** 10.3389/fgene.2024.1389095

**Published:** 2024-05-22

**Authors:** A. Rasim Barutcu, Michael B. Black, Raymond Samuel, Scott Slattery, Patrick D. McMullen, Andy Nong

**Affiliations:** ScitoVation, Durham, NC, United States

**Keywords:** new approach methodologies, transcriptomics, mode of action, benchmark dose, alternative splicing

## Abstract

Toxicological risk assessment increasingly utilizes transcriptomics to derive point of departure (POD) and modes of action (MOA) for chemicals. One essential biological process that allows a single gene to generate several different RNA isoforms is called alternative splicing. To comprehensively assess the role of splicing dysregulation in toxicological evaluation and elucidate its potential as a complementary endpoint, we performed RNA-seq on A549 cells treated with five oxidative stress modulators across a wide dose range. Differential gene expression (DGE) showed limited pathway enrichment except at high concentrations. However, alternative splicing analysis revealed variable intron retention events affecting diverse pathways for all chemicals in the absence of significant expression changes. For instance, diazinon elicited negligible gene expression changes but progressive increase in the number of intron retention events, suggesting splicing alterations precede expression responses. Benchmark dose modeling of intron retention data highlighted relevant pathways overlooked by expression analysis. Systematic integration of splicing datasets should be a useful addition to the toxicogenomic toolkit. Combining both modalities paint a more complete picture of transcriptomic dose-responses. Overall, evaluating intron retention dynamics afforded by toxicogenomics may provide biomarkers that can enhance chemical risk assessment and regulatory decision making. This work highlights splicing-aware toxicogenomics as a possible additional tool for examining cellular responses.

## Introduction

Genomic and transcriptomic approaches in toxicology provide unprecedented molecular insight into the mode-of-action (MOA) and derivation of points-of-departure (PODs) while reducing time, increasing throughput, and lowering the cost (Chepelev et al., 2015; Harrill et al., 2019; 2021; Baltazar et al., 2020; Franzosa et al., 2021). Thus, these approaches are becoming increasingly well-accepted as alternatives to conventional *in vivo* studies to derive PODs for assessing chemical safety in screening studies (Pagé-Larivière et al., 2019; Baltazar et al., 2020; Johnson et al., 2020; Pouzou et al., 2020). Recently, a focus of transcriptomics in toxicology has been the dose-response modeling of gene expression. This application couples benchmark dose (BMD) modeling to derive transcriptomic POD values determined by the gene expression changes induced by the chemical and gene ontology pathway analysis (EFSA Scientific Committee et al., 2017; Farmahin et al., 2017; Jensen et al., 2019). This approach identifies specific POD levels that are altered due to chemical exposure. The National Toxicology Program (NTP) report outlines an approach for genomic dose-response that has been effective for *in vivo* studies, where the biological effect POD closely approximates apical toxicology endpoints. For *in vitro* studies, additional steps like *in vitro* to *in vivo* extrapolation (IVIVE) modeling may be needed before the transcriptomic results can be effectively used for quantitative risk assessment (National Toxicology Program, 2018). Despite the advent of bioinformatic approaches and pipelines to determine the MOA and POD values of a chemical by transcriptomics, there is not yet any set consensus on data exploration. Therefore, it is crucial to conduct a systematic analysis of the bioinformatic approaches employed for deriving BMD and POD values.

The commonalities and differences observed in gene ontology pathways between chemicals can be attributed to their shared ability to induce oxidative stress, a well-documented mechanism in toxicology. For instance, pathways related to mitochondrial function, cell respiration, and DNA damage repair are often affected by oxidative stress as the cell attempts to counteract the damage caused by reactive oxygen species (ROS). Additionally, chemicals may target specific pathways or processes depending on their unique chemical structures and MOA. To further elucidate these relationships, additional studies and experiments are needed to provide a more detailed understanding of how oxidative stress mediates the observed gene ontology pathway changes in response to specific chemicals.

Alternative splicing is a fundamental cellular mechanism that enables a single gene to produce multiple distinct mRNA isoforms, thereby expanding proteomic diversity and regulating gene expression (Baralle and Giudice, 2017). Among the various types of alternative splicing events, intron retention, where introns are retained within the mature RNA, has garnered increasing attention due to its potential role in cellular processes and disease. While the significance of alternative splicing is well-established, the functional implications of intron retention remain relatively unexplored. Recent studies have begun shedding light on the regulatory roles of intron retention in diverse biological contexts, including neurodevelopment, immune responses, and cancer progression (Braunschweig et al., 2014; Wong et al., 2016; Jacob and Smith, 2017). The involvement of intron retention in toxicology and its potential as a molecular mechanism remain largely untapped.

In this study, we examine transcriptomics for short-term *in vitro* exposures with chemicals modulating cellular oxidative stress (Kappus, 1987). Oxidative stress is caused by an imbalance between the production and accumulation of ROS in cells and tissues and the ability of a biological system to detoxify these reactive products. ROS can be generated as by-products from the metabolism of environmental pollutants or xenobiotics that lead to imbalance and eventually tissue damage. Physiological and biological stress responses such as immune cell activation, inflammation, infection, cancer, or aging are all also responsible for the endogenous production of oxidative species. Excess ROS can adversely affect several cellular structures, such as membranes, lipids, proteins, lipoproteins, and DNA (Mittal et al., 2014). Chemical-induced oxidative stress represents an external influence on normal homeostatic processes and may exert effects at the transcriptome level differently for different chemicals that impact oxidative stress response.

Transcriptomic events occurring at doses prior to cell death can provide a clear insight into whether toxicity is an accumulation of cell damage or an acute response to high-dose exposure to a chemical. In an effort to investigate these possibilities, we examined five effectors of oxidative stress - diazinon, paraquat, prochloraz, sulforaphane, and 2,4-dinitrochlorobenzene (DNCB)—in a dose-response exposure using the A549 lung adenocarcinoma cancer cell line. The A549 lung adenocarcinoma cell line was selected because it is a well-characterized model that has been extensively used to study oxidative stress and chemical toxicity (Lukaszewicz et al., 2019; Upadhyay et al., 2019; Niechoda et al., 2023), and harbor functional cytochrome P450 enzymes and antioxidant response pathways that allow them to respond to and metabolize xenobiotics that modulate oxidative stress (Hukkanen et al., 2000). The chemicals were chosen for their varied mechanisms of impacting oxidative stress (Supplementaryu Table S1). Cell viability and RNA-seq experiments were performed using a nine-concentration dose-response experimental setup. The RNA-seq data were analyzed for differential gene expression, alternative splicing events, BMD modeling, and subsequent POD derivation following ontology pathway enrichment analyses. Taken together, these results presented in this study underscore the underutilized potential of intron retention in the context of toxicogenomics and its possible in illuminating MOAs for various chemicals. By comparing gene expression and alternative splicing events, this study examines the value of combining these tools by evaluating compounds with different characteristics that affect oxidative stress.

## Materials and methods

### Cell culture

A549 cells were obtained from the American Type Culture Collection (ATCC). Proliferation media consisted of Dulbecco’s Modified Essential Medium (Gibco) with 10% fetal bovine serum (FBS) (Atlanta Biologicals) and 1% penicillin-streptomycin (Gibco). The plating medium consisted of Dulbecco’s Modified Essential Medium (Gibco) with 10% Heat-Inactivated FBS (HI FBS; Atlanta Biologicals) and 1% penicillin-streptomycin (Gibco). For passaging, cells were washed with sterile Dulbecco’s Phosphate Buffered Saline (DPBS) and enzymatically removed using 0.25% trypsin-EDTA (Gibco). In preparation for the proliferation assay, A549 cells were thawed (2.0 x 106, passage 3) and placed into a T25 flask (United States Scientific). After 5 days in culture, cells were passaged into a T75 flask (2.0 × 106, passage 4) using a plating medium (10% HI FBS). After 5 days, cells were plated at a density of 35,000 cells/well in black-walled, clear-bottom, tissue-culture-treated 96-well plates (Greiner Bio-One) using a plating medium (10% HI FBS). Cells were cultured for 48 h in 96-well plates prior to treatment. Cell viability was measured using a bioluminescent assay for ATP in lysed cells (Promega G7571, CellTiter-Glo assay) with 3 biological and 3 technical replicates for each condition.

### Chemicals and reagents

Test chemicals, sulforaphane (Chemical Abstracts Service Registry number (CASRN) 4478-93-7), prochloraz (CASRN 67747-09-5), paraquat (CASRN 1910-42-5), diazinon (CASRN 333-41-5), and 2,4-dinitrochlorobenzene (DNCB - CASRN 97-00-7) were procured from Sigma. All chemicals had >98% purity, except sulforaphane, which had >90% purity. Stock solutions were made up of molecular biology grade (>99.9% purity) dimethyl sulfoxide (DMSO) (Sigma) or deionized water (paraquat and sulforaphane). The stock solutions were diluted into media prior to adding to cells, keeping the solvent concentration the same in all wells. Each compound was tested in eight concentrations in half-log increments from the highest dose tested. Diazinon, prochloraz, and DNCB were dissolved in DMSO, while paraquat and sulforaphane were dissolved in sterile deionized water. DMSO vehicle control wells contained 0.1% DMSO in culture medium, matching the final DMSO concentration in chemical treatment wells. Deionized water vehicle controls were used for paraquat and sulforaphane treatments. For serial dilutions, the chemicals were diluted using the same respective solvents to maintain consistent vehicle concentrations across all doses. All treatment wells contained 500 μL of cell culture medium. Chemicals or vehicle compounds were added in a 50 μL volume to achieve the desired final concentrations. Treatment plates were prepared fresh before each experiment to avoid evaporation issues.

### RNA-Seq data generation and analysis

Using 4 biological replicates for each condition, whole cell RNA-seq was performed by Novogene Corp. using polyA enrichment. The RNA isolation was performed by utilizing the RNeasy Micro Kit (Qiagen Cat. No. 74004) with polyA enrichment. The retained RNA underwent quality assessment with a RIN >9 threshold, and purity was verified based on absorbance ratio measurements (260:280 nm ratio of −2). Illumina 150bp paired-end read kits (TruSeq Stranded mRNA Library Prep Kit (Cat #20020594) as per the instructions supplied with the kits. Reads were mapped to the Ensembl human reference genome (hg38) using HISAT2 (version 2.05) (Zhang et al., 2021). Each biological replicate of each condition was sequenced to an average depth of −40 million reads (standard deviation −5.2 million reads). Preceding the mapping step, reads were subjected to trimming and quality filtering processes to enhance data accuracy and reliability. RNA-seq results were uniformly of high quality (>90% mapping rate). The replicates displayed high QC quality with regards to the mapped count data and correlation, and thus each concentration for every chemical was analyzed using all four replicated samples. The 4 biological replicates displayed high QC quality with regards to the mapped count data and correlation, and thus all 4 replicates for every condition were analyzed using DESeq2 for differential gene expression and VAST-tools for alternative splicing analysis. FeatureCounts (Liao et al., 2014), a part of the Subread package, was employed to quantify the read counts for each gene from the aligned RNA-seq data. The analysis involved specifying the aligned BAM files as input, and the resulting count matrix was utilized for downstream expression analysis.

### Differential gene expression analyses

The open-source statistical program R was used to run the DESeq2 library (Love et al., 2014), and normalized log2 (gene count+1) transformed data for BMD analyses were generated with DESeq2 for all analyses. Genes that harbored less than 10 gene counts were removed from the dataset prior to DeSeq2 analysis. To capture genes altered by exposure to compound with high confidence, we applied corrected *p*-value (false discovery rate) of less than 0.05 (FDR<0.05) and a fold change greater than 1.5-fold (|FC|>1.5). The Venn diagrams were generated by using the DeepVenn package (Hulsen, 2022). The gene ontology analysis was performed with the gProfiler software (Kolberg et al., 2023).

### Analysis of Splicing events

Alternative splicing analysis of RNA-Seq data was performed with VAST-Tools (version 2) (Tapial et al., 2017), a comprehensive software suite designed for the identification and quantification of various alternative splicing events from RNA-Seq data. VAST-Tools employs a rigorous methodology that aligns the reads to the reference genome and transcriptome, accurately identifying splice junctions and quantifying alternative splicing events, including intron retention, exon skipping, alternative 5′and 3′splice site usage, and mutually exclusive exons.

To identify differentially regulated alternative splicing events across the different chemical treatments and doses, we utilized the “diff” module of VAST-Tools. This module compares the Percentage Spliced In (PSI) values, which represent the inclusion ratio of a particular alternative splicing event, between the treatment and control conditions. We then applied a threshold of |dPSI| > 5%, which means that only alternative splicing events with an absolute difference in PSI values greater than 5% between the treatment and control conditions were considered significant.

Additionally, we employed the MV_dPSI_at0.95CI > 0 criterion, which ensures that the 95% confidence interval for the mean difference in PSI values removes non-confident events. This step further enhances the statistical robustness of the analysis by accounting for biological variability and ensuring that the observed differences in alternative splicing are statistically significant.

By combining these two criteria, we identified alternative splicing events that were consistently and significantly altered across the different chemical treatments and doses. The raw codes and usage of VAST-tools can be obtained from https://github.com/vastgroup/vast-tools.

### Benchmark dose modeling

The BMDExpress2 package was used to identify different BMD models for the ontology enrichment genes and pathways (Phillips et al., 2019). A complete description of the BMD modeling approach was also previously described (Black et al., 2022). All models were fitted assuming constant variance as the data were log2 transformed after the normalization in DESeq2. A final best fitting model was determined by first determining the best fitting polynomial model by nested Chi square test. The best fit polynomial model was then compared to the remaining models to select a final overall best fitting model by Akaike information criteria (AIC). A BMD factor of 1 standard deviation (SD) was used. The option for flagging the Hill model with “k” parameter less than ⅓ of lowest positive dose was utilized, and best model selection with flagged Hill model was done by selecting the next best model with *p*-value >0.05. First, any model with extrapolated BMD value beyond the highest or the lowest concentrations used in the dose-response experiment was rejected. Similarly, any best fitting model with a goodness of fit *p*-value less than 0.1 was rejected. Finally, any model which had a BMDU/BMDL ratio greater than 40 was rejected to avoid BMD estimates with excessively large 95% confidence intervals.

Ontology enrichment was performed using the publicly available GO ontology database. BMDExpress2 performs a conventional over-representation test of the query gene lists relative to the pathway annotated gene lists in Gene Ontology. For BMD analyses, the data were prefiltered using the ANOVA test (*p* ≤ 0.05) and a fold-change of ≥1.5 or ≤1.5. Data were then filtered with the settings in BMDExpress v2.3 of best BMDU/BMDL <40, and best fitPvalue >0.1. We set a significance threshold for the enrichment of a pathway having at least 5 of our query genes found amongst the pathway elements, and with a Fisher’s Exact test (two-tailed) *p*-value from the over-representation test that was less than 0.05. A pathway BMD value is computed as the median gene based BMDU/BMD/BMDL values for the genes found amongst the pathway category elements.

For intron retention BMD modeling, we used the number of transcript-mapped reads to calculate the expression levels of individual introns, and they were modeled at the “intron” level in BMDExpress2, similar to the approach for gene expression counts. Specifically, the intron retention mapped read counts were log2 transformed and median-normalized per sample prior to BMD modeling. This matches the preprocessing for gene counts. After the BMD calculation step, the corresponding genes were used to perform the BMD pathway analyses.

Significantly enriched pathways and computation of pathway-based POD values were obtained by selecting genes from the ontology enrichment analyses. For ontology enrichment results, we summarized the 20 most sensitive enriched Gene Ontology (GO) pathways (*p* < 0.05, genes that passed all filters >5). Comparisons were restricted to median pathway values or averages of the median BMD values of genes with a pathway as our experience with other data (not shown) has shown these are consistently more conservative than pathway mean values.

The GO analysis presented in Figure 2 was conducted based on data from single dose concentrations, involving the categorization of genes as either up- or downregulated. This initial analysis aimed to capture early molecular responses to the various chemicals studied. In contrast, the subsequent BMD pathway analysis, conducted using BMDExpress2, took a different approach. It centered on calculating the increase or decrease in gene expression that aligns with one of the mathematical models tested, thereby providing a more comprehensive representation of dose-response relationships across the entire concentration range. By utilizing BMDExpress2, this analysis aimed to identify critical benchmark concentration values (BMDL, BMD, and BMDU) that serve as thresholds indicative of the chemicals’ impact on molecular pathways. This transition from single dose GO (molecular, biological, and functional domains) analysis to BMD pathway analysis generates a deeper and more nuanced understanding of the molecular changes induced by these oxidative stressors. The network analysis was performed by using the CytoScape software (v3.9.1).

## Results

### Cell viability of A549 cells to different oxidative stress inducers

**FIGURE 1:**
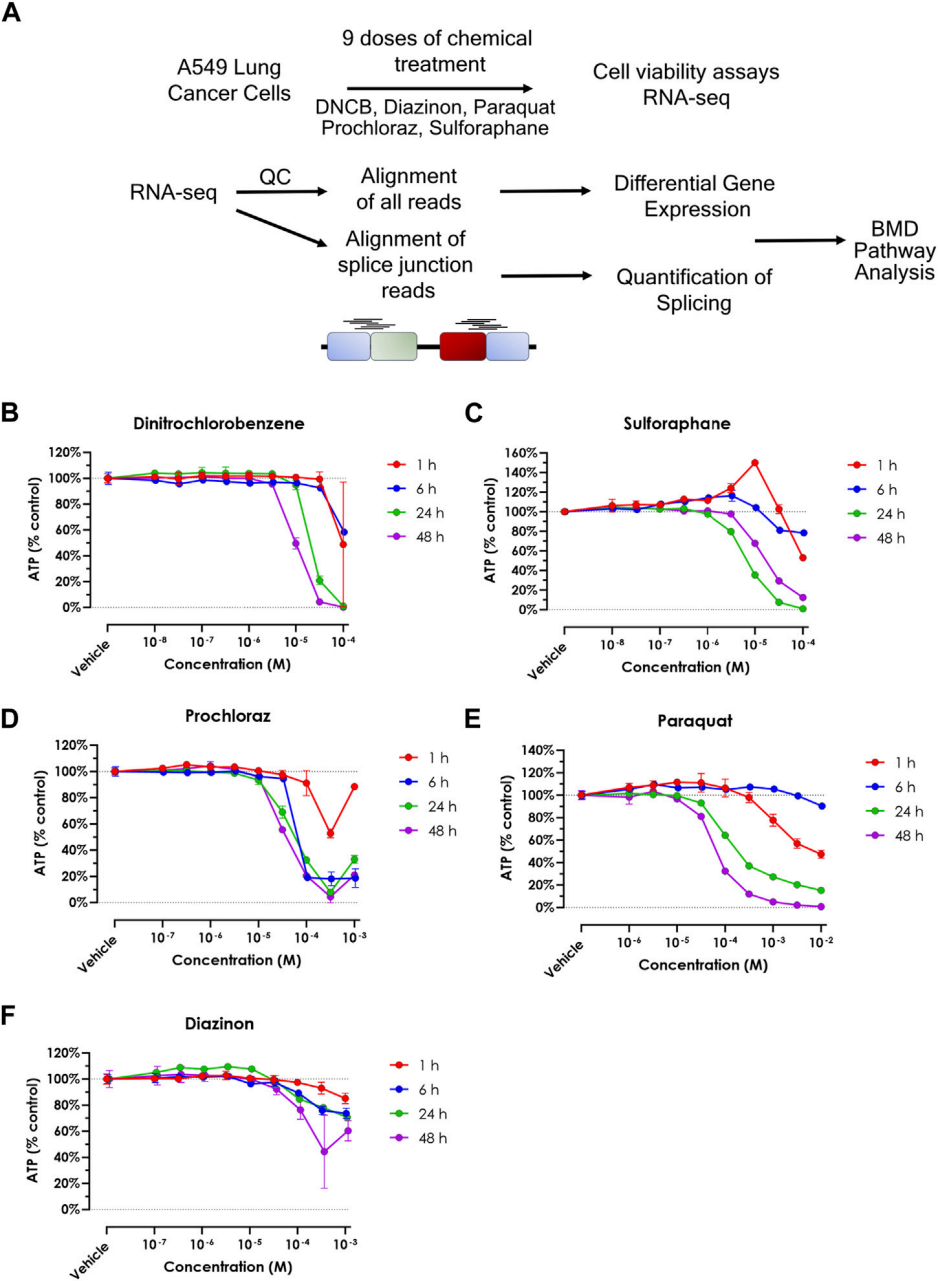
Cell viability upon treatment of oxidative stress modulators. **(A)** Schematic overview of the experimental procedure in this study. **(B–F)** A549 cell viability assay for **(A)** DNCB, **(B)** Sulforaphane, **(C)** Prochloraz, **(D)** Paraquat and **(E)** Diazinon. Blue = 1 h, red = 6 h, green = 24 h and purple = 48 h.

In this study, we assessed 5 oxidative stress effectors using toxicogenomics analyses (Figure 1A, Supplementary Table S1). To assess the sensitivity of the A549 cell line to each chemical, and to determine the concentration range for transcriptomic studies, we performed cell viability assays (Figures 1B–F). This experiment allowed us to identify the upper concentration for each of the five chemicals, where the concentrations for the transcriptomic experiments consisted of half-log dilution series from that maximum concentration. These values were 10-4 M for Paraquat and Prochloraz, 10-5 M for Sulforaphane and DNCB, and 10-3 M for Diazinon (Figures 1B–F). For RNA-seq experiments, the maximum concentration, where the cell viability decreased by 15%–25%, was selected to prevent confounding cell death and cytotoxicity and the loss of viable RNA. The 24-h exposure was chosen as the time point since this time point displayed intermediate effects when compared to shorter and longer time points. Altogether, these experiments determined the viability rates of A549 cells upon exposure to different oxidative stress inducers and allowed us to choose the optimum dose for dose-curve transcriptomics experiments.

### RNA-seq and differential gene expression

**FIGURE 2:**
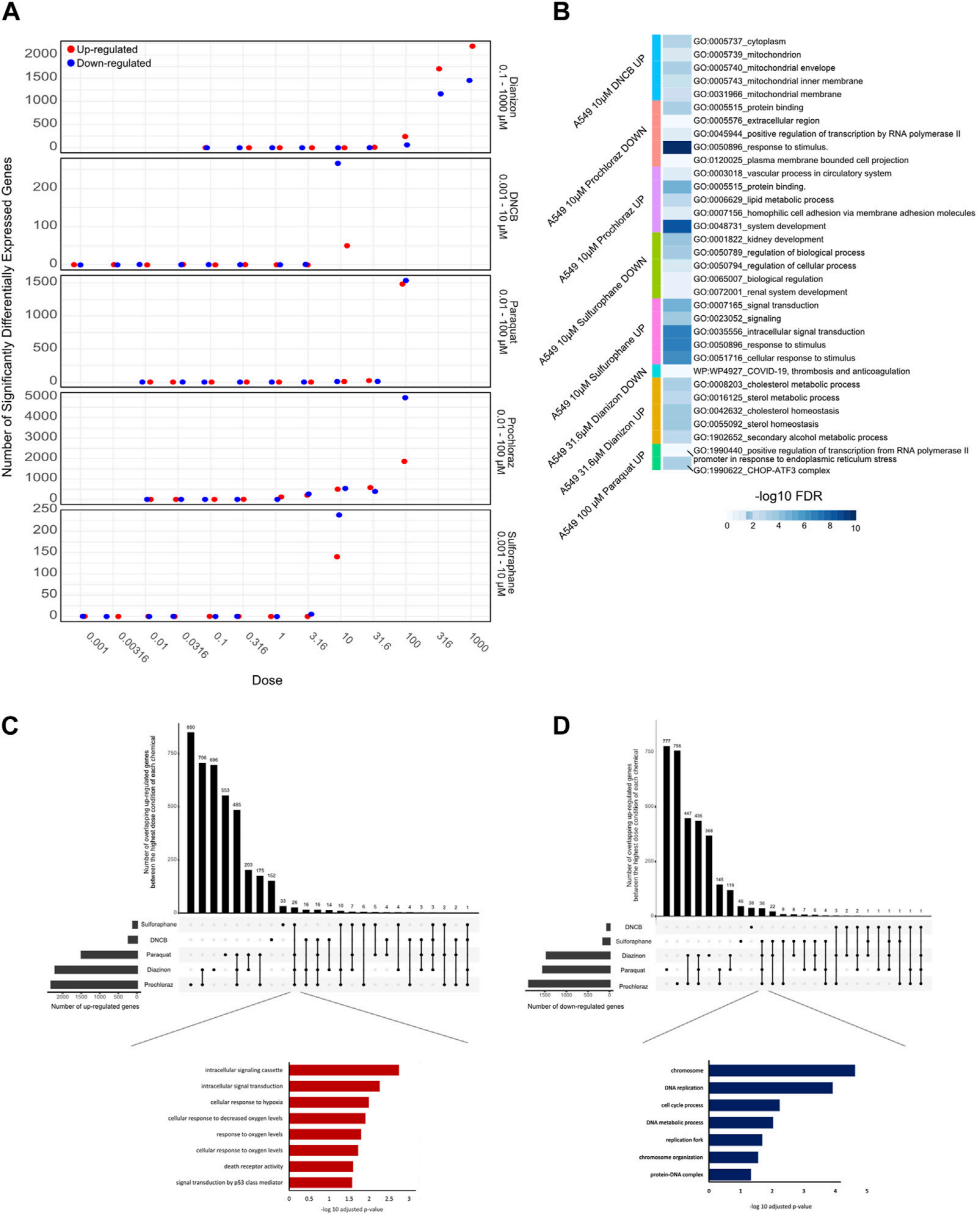
Transcriptomic analysis of oxidative stress modulators. **(A)** Dot plot showing the number of upregulated (red) and downregulated (blue) genes for the 5 chemicals across the doses tested. **(B)** Heatmap showing the -logFDR value of the gene ontology pathways for the lowest chemical concentrations in which there was observed differential gene expression. **(C)** Upset plot showing the number of upregulated genes at the highest dose concentration for each oxidative stress modulator. The bars on the left represent the total number of upregulated genes for each compound, while the bars on top indicate the number of genes shared among different combinations of compounds. The inset shows the enriched Gene Ontology (GO) terms for the overlapping upregulated genes across most compounds, highlighting their association with oxidative stress response, and signaling pathways. **(D)** Upset plot showing the number of downregulated genes at the highest dose concentration for each oxidative stress modulator. The inset shows the enriched Gene Ontology (GO) terms for the overlapping downregulated genes across most compounds, highlighting their association with DNA metabolism, cell cycle processes, and chromosome organization.

RNA-Seq results were uniformly of high quality with an average of −40 million mapped reads per replicate. The replicates displayed high QC quality with regards to the mapped count data and correlation, and thus each concentration for every chemical was analyzed using all 4 replicated samples. Differential gene expression results (see Methods for details) for A549 cells show that at highest dose concentration, there were hundreds to thousands of differentially expressed genes (DEGs) for all five chemicals. We observed a clear dose-response of DEGs with diazinon and prochloraz with the A549 cells (Figure 2A; Table 1).

**TABLE 1:** The number of genes dysregulated for each condition.

Diazinon (µM)	0.1	0.316	1	3.16	10	31.6	100	316	1000
Upregulated	0	0	0	0	0	9	242	1702	2190
Downregulated	0	0	0	0	0	2	60	1162	1451
DNCB (uM)	0.001	0.00316	0.01	0.0316	0.1	0.316	1	3.16	10
Upregulated	0	0	0	1	0	0	0	0	50
Downregulated	0	0	1	0	0	0	0	1	266
Sulforaphane (uM)	0.001	0.00316	0.01	0.0316	0.1	0.316	1	3.16	10
Upregulated	0	0	0	0	0	0	0	0	140
Downregulated	0	0	0	0	0	0	0	5	238
Prochloraz (uM)	0.01	0.0316	0.1	0.316	1	0.316	10	31.6	100
Upregulated	0	0	0	4	119	203	499	577	1862
Downregulated	1	1	0	0	0	264	535	389	4972
Paraquat (uM)	0.01	0.0316	0.1	0.316	1	0.316	10	31.6	100
Upregulated	0	0	0	0	0	0	0	22	1483
Downregulated	0	0	0	0	0	0	0	10	1536

Next, in order to assess the pathways that were altered via the treatment of each chemical, we performed GO analysis of DEGs identified with the lowest dose concentration in which significantly differentially expressed genes and pathways were identified for each compound and cell line (Figure 2B).

The 31.6 µM diazinon treatment in A549 cells revealed alteration of genes involved in GO terms such as the “apolipoprotein binding” or “cholesterol homeostasis”. 10 µM DNCB treatment revealed pathways such as “respiratory pathway IV” and “mitochondrial envelope” in A549 cells, indicating the perturbation of respiratory pathways. Next, GO analysis of 10 µM sulforaphane treatment revealed the perturbation of “protein binding” or “cell surface” pathways. The lowest doses of which prochloraz treatment showed a DEG and perturbed pathways were 10 µM for A549 cells. GO terms altered in A549 cells in this condition included “circulatory system process” “transcriptional activity” and “cell-matrix adhesion”. Finally, 31.6 µM paraquat treatment in A549 cells revealed pathways such as “long chain fatty acid metabolism” or “signaling receptor binding” in A549 cells (Figure 2B).

In summary, these findings indicate that examining lower doses where DEGs are observed unveils diverse pathways affected. An exception to this would be DNCB, which led to aberrant expression of genes within the oxidative respiratory pathway. We identified upregulation of expected oxidative stress marker genes, such as SOD1, HMOX1 and NQO1, and relevant pathways related to oxidative stress responses. Therefore, the differential gene expression and GO pathway analysis using transcriptomics reveal overlapping as well as differing number of DEGs and enriched molecular, cellular and functional GO pathway categories across the 5 oxidative stress modulators, providing a highly detailed information about the molecular changes associated with each compound (Figure 2B).

To analyze the common cellular responses between the chemicals, we assessed the extent of commonly regulated up- and downregulated genes in the highest dose condition of each chemical that are unique or shared among the different compounds (Figures 2C,D). A substantial number of genes were commonly upregulated across multiple chemicals, with a core set of 26 genes shared by all compounds except DNCB (Figure 2C). GO analysis of the overlapping upregulated genes revealed a significant enrichment of terms associated with oxidative stress response, such as “cellular response to oxygen levels,” “cellular response to decreased oxygen levels,” and “cellular response to hypoxia” (Figure 2C, inset). In contrast, the analysis of overlapping downregulated genes (Figure 2D) revealed 36 genes that commonly overlap across all compounds except DNCB. GO analysis of the overlapping downregulated genes highlighted terms related to DNA metabolism, cell cycle processes, and chromosome organization (Figure 2D, inset), indicating potential disruptions in these cellular processes.

### Benchmark concentration modeling and POD derivation

To gain insight into the pathways that are affected as a function of increasing dose concentrations, we used BMD analyses to derive POD values from the transcriptomic data of each chemical. Given the varying approaches found in the literature (Harrill et al., 2021; Matteo et al., 2023) and the evolving nature of transcriptomics data analysis, we chose to conduct a comprehensive and impartial analysis by employing two distinct methods. First, we applied a significance criterion for BMD pathways, where each pathway contained more than 5 genes and displayed an FDR value of less than 0.05 (see Methods) and selected the 20 lowest BMD pathways. For the second method, we analyzed the BMD values for individual intron retention events.

**FIGURE 3:**
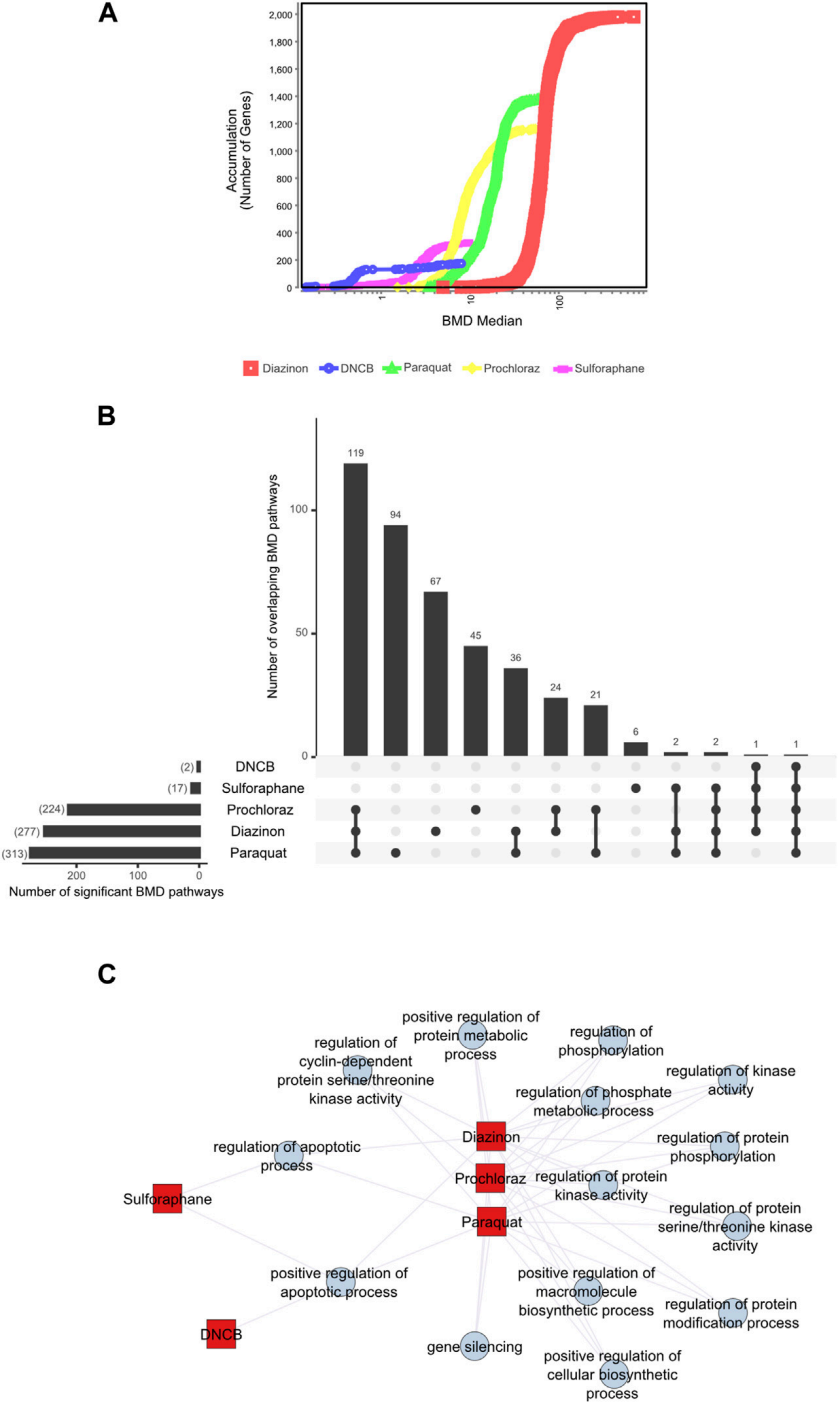
BMD analysis using gene expression. **(A)** BMD Median Accumulation plot for the genes in each tested condition. The ANOVA test (*p* < 0.05) and a fold-change of >1.5 or < −1.5 were used to pre filter the data. In BMDExpress v2.3, data were also post-filtered for best BMDU/BMD 40 and best fitPvalue >.1. **(B)** Upset plot showing the number of BMD pathways that are either unique to each condition or overlapping with multiple conditions. The lines that connect the dots below indicate an overlap between the conditions, and the bar graph on top indicates the number of overlapping pathways. The bar graph on the left indicates the number of observed BMD pathways for each condition. **(C)** Network graph showing the connectivity of the BMD pathways that display the highest rate of overlap among the tested conditions.

First, we applied a significance criterion for BMD pathways, where each pathway contained more than 5 genes and displayed an FDR value of less than 0.05 (see Methods) and selected the 20 lowest BMD pathways. This approach led to hundreds of pathways being identified (Figure 3B). Analysis of the average lower benchmark dose (BMDL), BMD and upper benchmark dose (BMDU) values of the lowest 20 pathways revealed values that were consistent with the cell viability assays (Figure 1). Since only sulforaphane and DNCB had two enriched pathways with A549 cells, a single summary POD was derived based on the median value for all pathways for these results. The average BMDL values for the 20 most significant pathways were compared to the average BMDL values for all significant pathways. In all cases, the average BMDL for the 20 most significant pathways was lower than the average BMDL for the full set of significant pathways. We observed BMDL values of 19.9, 3.3, 2.43, 0.5, and 0.29 μM for diazinon, paraquat, prochloraz, DNCB, and sulforaphane, respectively. As a result, this analysis highlighted several known features of these compounds and unearthed unexpected MOA’s (see Discussion).

Next, we visualized the BMD accumulation plots for genes that exhibited responses below the maximum tested dose for each condition (Figure 3A). A549 cells treated with DNCB displayed the gene distribution with the lowest BMD accumulation, whereas cells treated with diazinon had the highest (Figure 3A). We next analyzed the BMD pathways that were commonly shared among all the chemical conditions and identified that pathways such as “regulation of serine/threonine kinase activity” or “metabolic” and “apoptotic process” that were overlapping (Figures 3B,C, Supplementary Figure S2). A wide range of cell cycle and DNA damage endpoints were most predominant among the pathways observed at the low concentration range. Notable cellular responses to oxidative or chemical stress pathways were apparent in cells exposed to diazinon, paraquat, prochloraz, and DNCB at the BMD and BMDL levels observed. Only paraquat demonstrated major chemical oxidative stress response pathways observed.

Our analysis highlights shared toxicity pathways while also revealing some substantial differences between the chemicals (Supplementary Figure S2). Core cell cycle control and DNA damage pathways showed sensitivity to all compounds, implying general reactive toxicity. However, paraquat uniquely disrupted MAPK signaling (Liu et al., 2022), potentially reflecting oxidative inhibition of phosphatases. Diazinon’s impacts on immune pathways align with its acetylcholinesterase inhibition (Colović et al., 2013). Sulforaphane and DNCB showed earlier perturbation of translation and RNA processing. Prochloraz’s low-dose effects on reproductive pathways match its endocrine disruption (Vandenberg et al., 2012). Thus, despite common downstream outcomes like cell cycle dysfunction, signatures of distinct molecular initiating events emerged. Overall, combined assessment of shared and unique BMD-perturbed pathways facilitates MOA elucidation, aiding chemical characterization and risk analysis.

These analyses altogether highlight the specific and shared pathways that are perturbed with each chemical and underscore the importance of careful BMD analysis pipelines for the identification of MOAs.

### Alternative splicing analysis

**FIGURE 4:**
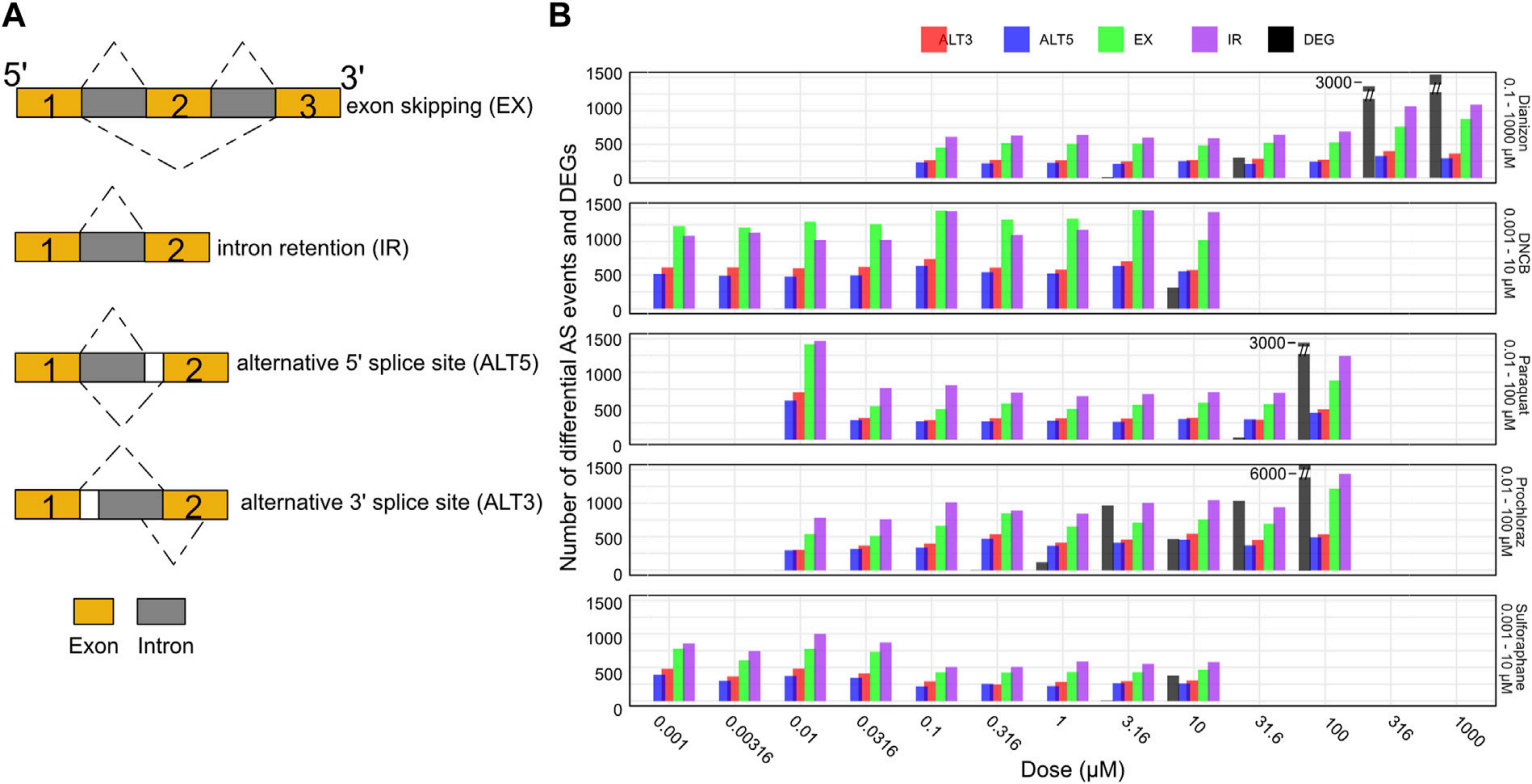
Splicing changes occur prior to changes in gene expression. **(A)** Schematic representation of the major alternative splicing events observed. **(B)** Bar plot showing the number of exon skipping (EX), intron retention (IR), alternative 3′ splice site (ALT3), and alternative 5′ splice site (ALT5) events along with the number of differentially expressed genes (DEG) for the five chemicals across the doses. The splicing events are observed at concentration where there aren’t any DEGs.

Alternative splicing generates multiple mRNA isoforms from a single gene through mechanisms like exon skipping, intron retention, and alternate splice site usage (Figure 4A), allowing individual genes to produce multiple transcript isoforms, expanding proteomic diversity (Blencowe, 2006; Lee and Rio, 2015; Tapial et al., 2017; Park et al., 2018; Ule and Blencowe, 2019). This process is regulated by splice sites, splicing factors, and RNA-binding proteins that control exon inclusion/exclusion and intron excision. Major splicing patterns include cassette exon skipping, intron retention, and use of alternate 5′/3′ splice sites (Figure 4A). Alternative splicing can influence the relative abundance of different transcript isoforms originating from the same gene, thereby impacting the composition of the transcript pool without necessarily altering the overall gene expression levels. Despite its prevalence in eukaryotes, and its frequent perturbation in several developmental diseases and cancer (Scotti and Swanson, 2015), the role of alternative splicing in toxicology remains under-explored (Zaharieva et al., 2012; Villaseñor-Altamirano et al., 2019; Banerjee et al., 2020).

Here, we utilized the RNA-seq data to systematically investigate splice variants associated with each chemical (see Methods) using the widely utilized VAST-Tools software (Tapial et al., 2017). VAST-Tools quantifies alternative splicing events by counting RNA-seq reads mapping uniquely to splice junctions and involved exons, then calculates an inclusion ratio for each event representing the relative abundance of isoforms with or without the event. Alternative splicing analysis revealed extensive changes in events such as exon skipping (EX) and intron retention (IR) across the tested chemicals. All chemicals elicited robust changes in the four splicing event types often at low doses (Figure 4B, Supplementary Figure S3A). For instance, diazinon triggered the inclusion of a high number of IR and EX events by 0.316 μM, preceding toxicity. DNCB similarly resulted in marked IR and EX changes at 0.001 μM, below concentrations impairing viability or gene expression. Most compounds displayed preferential modulation of certain event types, illustrating the complexity of splicing regulation. However, IR and EX were consistently the predominant response, emerging as generalizable markers of chemical perturbation. In contrast, ALT3 and ALT5 events were detected in lower numbers. Thus, it may be possible that all types of splicing events could be playing a combinatorial role in oxidative stress. While many chemicals elicited splicing perturbations without significant differential expression, sulforaphane uniquely induced gene expression alterations alongside splicing effects. Overall, alternative splicing analysis provides another window to query chemical effects overlooked by standard expression profiling.

### Benchmark concentration modeling using intron retention events

Of the splicing alterations elicited by chemical toxicants, intron retention (IR) and exon skipping (EX) events were the most common splicing alterations across all compounds tested, highlighting it as a possible marker of cellular stress (Vu et al., 2022). Though under-appreciated relative to exon skipping, IR is now recognized to play key regulatory roles, influencing mRNA localization, stability, translation, and protein production (Wong et al., 2016; Jacob and Smith, 2017; Monteuuis et al., 2019). Retained introns introduce premature termination codons, enabling rapid message turnover. Moreover, the unique sequences and motifs within retained introns can affect splicing, RNA localization, transcription, and translation via RNA-binding proteins and chromatin modifiers.

**FIGURE 5:**
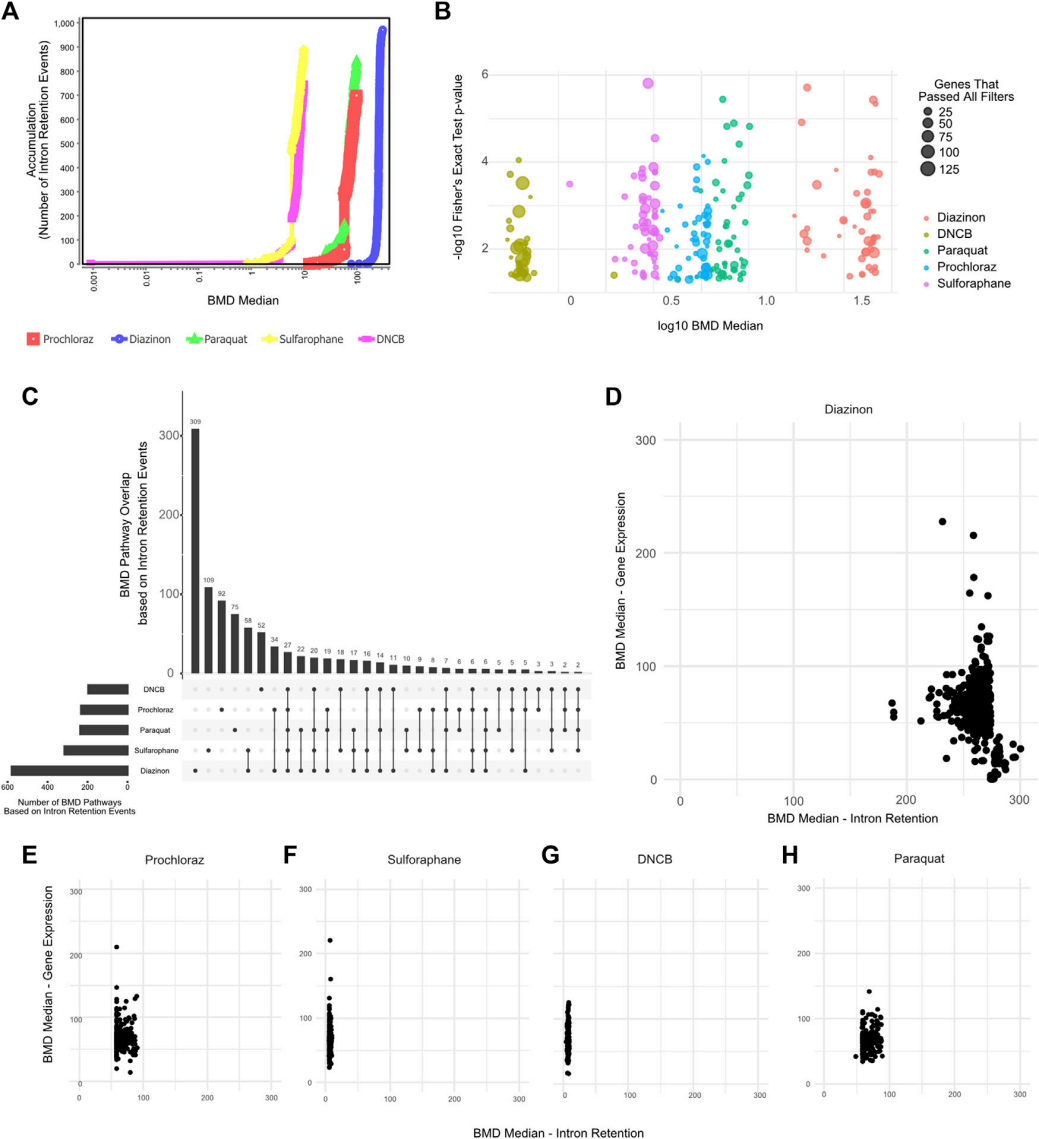
BMD Analysis using intron retention events. **(A)** BMD Median Accumulation plot, generated by using the intron retention events, for each tested condition. **(B)** Bubble plot showing the minus log10 *p*-value of lowest 50 BMD pathways as a function of Median BMD levels. **(C)** Upset plot showing the overlap of BMD pathways determined by intron retention events. **(D–H)** Scatter plot showing the Mean BMD values that have been generated either by gene expression profiling (*y*-axis) or splicing analyses (*x*-axis) for **(D)** Diazinon, **(E)** Prochloraz, **(F)** Sulforaphane, **(G)** DNCB, and **(H)** Paraquat.

We therefore leveraged IR profiles to model dose-response relationships and benchmark concentrations. The BMD pathway accumulation plot revealed numerous intron retention events surpassing benchmark response cutoffs, even at low concentrations (Figures 5A,B). For instance, critical mRNA processing pathways emerged at a BMD of −4 μM for several of our test compounds. Upset plot analysis highlighted extensive overlap in BMD pathways across chemicals, centered on key processes like splicing and translation (Figure 5C). Interestingly, except for diazinon, the mean BMDs derived from intron retention events were lower than those from differential expression for the same compounds (Figures 5D–H). Accordingly, the number of BMD pathways detected by gene expression profiling partially overlapped with BMD pathways detected by IR profiling (Supplementary Figures S3B–F). This supports intron retention’s sensitivity, capturing pathway perturbations undetected by expression profiling. Overall, applying benchmark concentration modeling to global intron retention patterns revealed unexpected pathways and POD. Splicing-derived BMD modeling expands the utility of toxicogenomics.

The BMD analysis uncovered biological pathways and points of departure from IR that differed from those associated with gene expression. Our results reveal retained introns as promising targets meriting extensive analysis to elucidate chemical MOAs and enhance risk assessment.

**FIGURE 6:**
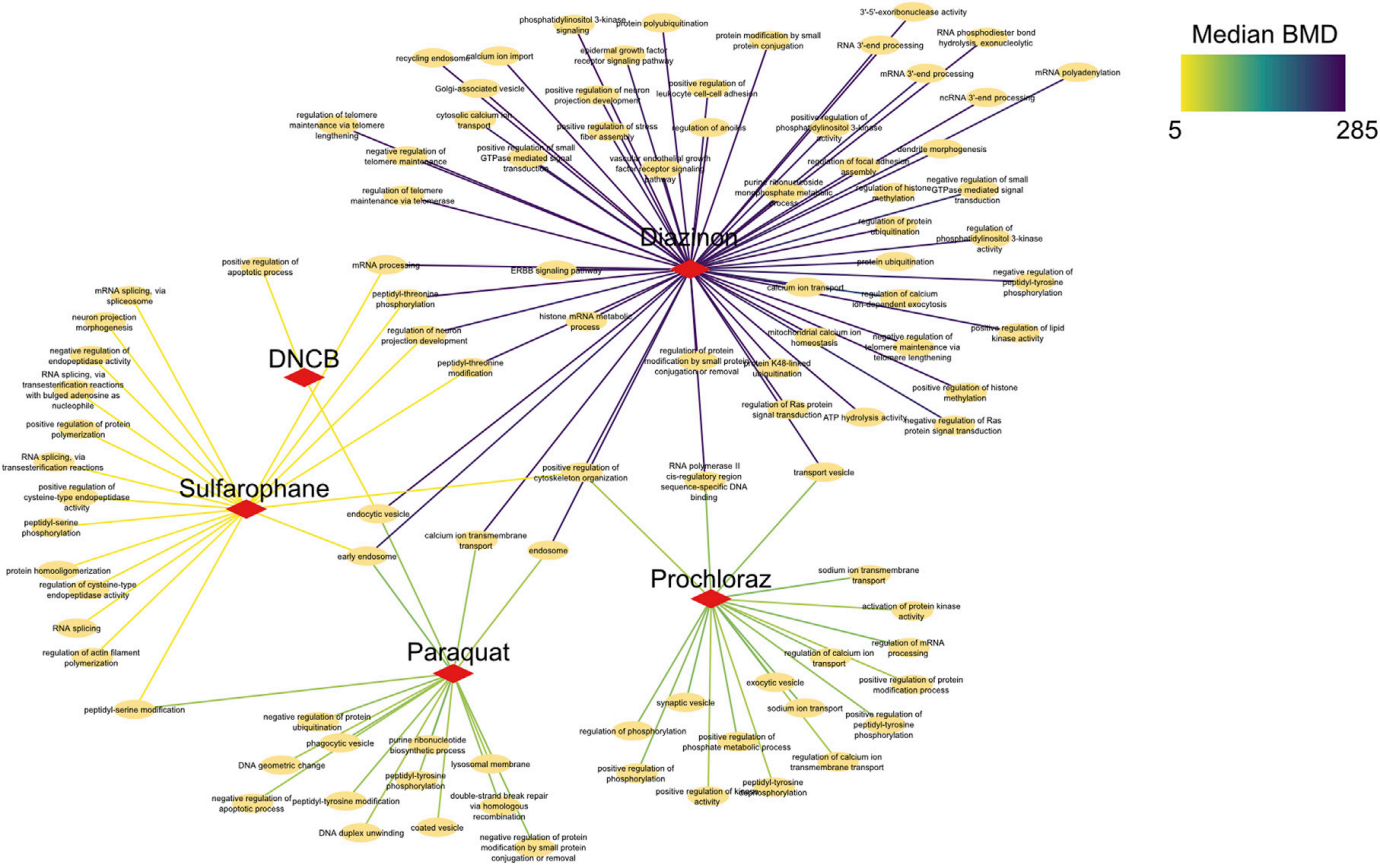
Network analysis of BMD pathways determined by intron retention events. Network graph showing the unique, as well as overlapping BMD pathways that have been determined by intron retention event modeling. The color of the edges indicates the Median BMD values.

To elucidate shared and unique biological changes across chemicals, we further constructed a pathway network to visualize the overlapping BMD pathways (Figure 6). Network visualization of pathways modulated at benchmark concentration levels revealed extensive connectivity and shared biology across the mechanistically distinct chemicals. Numerous pathways were jointly perturbed by multiple compounds, centered on key processes like mRNA processing, vesicle trafficking, and post-translational modifications. For instance, critical splicing and polyadenylation pathways were common to prochloraz, DNCB, and sulforaphane, three otherwise divergent oxidative toxicants. Similarly, regulation of ubiquitination and transport vesicles emerged as conserved hubs, suggesting generalized cellular stress effects. Furthermore, DNA repair pathways unique to sulforaphane emerged at higher BMD levels, providing chemical-specific insights. However, closer examination also revealed chemical-specific interactions reflecting unique alterations, such as DNA repair for sulforaphane or neuron development for DNCB. In addition, calcium signaling pathways showed lower median BMDs for DNCB *versus* prochloraz, implying greater potency despite common modulation. This network approach enhances compound-specific potency assessments, elucidating unique molecular initiation events and their dose-dependencies.

Collectively, these results demonstrate that combining BMD modeling with network biology provides both a broad overview of common toxicological mechanisms, as well as nuanced insights into the precise pathway perturbations underlying each compound’s toxicity. This systems-level perspective enhances molecule-specific risk assessment.

## Discussion

In this study, we sought to identify the pathways affected in a dose response manner by various oxidative stress modulators. The range of BMD values obtained for the chemicals showed a correlative trend with the doses where ∼15–25% cell death occurred in viability experiments (Figure 1). This trend is consistent with the fact that the DEGs also followed the same trend as the viability assays (Figure 2A). GO analysis using fixed, early dose concentrations revealed several mitochondrial and cellular respiration-associated pathways consistent with the known effects of these chemicals (Figure 2B).

This study demonstrates the potential of combining gene expression and splicing analysis to elucidate chemical mechanisms of action (MOAs) and PODs. While expression profiling revealed overt toxicity thresholds, systematic splicing characterization uncovered lower-dose RNA processing disruption, affirming RNA homeostasis as a toxicological MOA. Remarkably, intron retention emerged as the primary splicing alteration, providing a generalized indicator of cellular stress across structurally diverse toxicants, as also suggested by earlier reports (Hadar et al., 2022; Vu et al., 2022). By modeling dose-dependent intron retention profiles, we extracted benchmark concentration pathways and PODs at lower concentrations than those affecting gene expression. The minimal, but significant overlap between expression- and splicing-derived pathways affirms retained introns as informative, complementary biomarkers. Rather than superseding expression results, splicing analysis may add useful information.

The modulation of splicing events, encompassing intron retention, exon skipping, and 3′or 5′splice site selection, plays important roles in gene expression regulation (Jacob and Smith, 2017). Perturbations induced by environmental factors or toxicants can lead to aberrant splicing patterns, generating diverse mRNA isoforms, some of which may encode dysfunctional proteins that disrupt cellular processes. Altered splicing can also result in the accumulation of unstable RNA species, triggering stress responses. Moreover, changes in splicing may yield protein isoforms with varying activities and interactions, contributing to cellular dysfunction and toxicity. These alterations serve as valuable biomarkers, offering insights into the molecular mechanisms underlying toxicity and providing early indicators for comprehensive risk assessment in toxicology.

Our integrated transcriptomics approach provides broad, pathway-level insights through expression analysis, supplemented by event-level perturbations from splicing. By fusing both techniques, we obtained a more complete perspective into chemical MOAs, capturing low-abundance anomalies antecedent to downstream endpoints. Both techniques provide complementary insights. Gene expression defines chemical impacts on functional protein pathways that influence cellular outcomes. Meanwhile, splicing analyses reveals the fine-tuned regulatory disruptions underlying these transcriptomic shifts. Integrating both is crucial for a mechanistic understanding of how chemicals initiate molecular perturbations that propagate into overt toxicity. Future work should balance sensitive pathway examination with systems-level perspectives and investigate whether changes in alternative splicing is a hallmark within different toxicological contexts.

We identified intricate dose-dependent modulation of alternative splicing events (Supplementary Figure S3). With Diazinon and Sulforaphane, there is a balanced up- and downregulation of IR and EX events across all the doses. DNCB induces a robust downregulation of IR and EX events across all doses. Paraquat treatment leads to IR events that are consistently upregulated especially at lower doses, with balanced distribution at higher doses. Moreover, in the context of Prochloraz treatment, there is a dose-dependent decrease in the number of upregulated and an increase in the number of downregulated IR and EX events. These observations highlight the complex interplay between different splicing events and their dose-dependent modulation, which may be influenced by the specific mechanisms of action and cellular responses elicited by each oxidative stress modulator. The clear differences in the patterns of up- and downregulation across compounds underscore the need for a comprehensive analysis of alternative splicing events to fully understand the splicing dysregulation associated with oxidative stress and its implications for cellular function, toxicity and risk assessment. Comparison of altered pathways detected by differential gene expression and IR analysis reveals several common pathways that are co-regulated by these processes (Supplementaryu Table S4).

Differential basal expression of genes and pathways involved in mediating or counteracting chemical MOAs likely contributes to cell-type specific susceptibility (Black et al., 2023). Furthermore, compounds may preferentially disrupt pathways that are highly expressed in certain cell contexts due to tissue-specific roles, while not affecting cell types lacking associated gene programs. Elucidating how chemical perturbations interface with cell-intrinsic expression landscapes will shed light on selective sensitivities and improve cross-cell-type extrapolation.

Assessing significant BMD pathways which were commonly identified to be overlapping among the cell line-chemical combinations revealed that these pathways do not necessarily harbor the lowest BMD or BMDL (i.e., BMDL = POD) levels but are shared among the oxidative stressors and the 2 cell lines used (Figure 3). These analyses revealed several converging pathways that include metabolic processes, phosphorylation of proteins (i.e., serine/threonine kinases in cell cycle control) or regulation of gene expression by gene silencing. A detailed, gene-level BMD analysis of the top three overlapping pathways (Figure 3) indicates that there is a range of genes with differing BMD values that drive the alterations of these pathways.

Intron retention-based BMCs tended to be lower than viability or gene expression-based BMCs across most chemicals. However, there is a high correlation between the median BMC values of lowest BMC pathways and those that have been derived from cell viability assays (Krebs et al., 2020) (Supplementary Figure S4). This highlights the sensitivity of splicing alterations like intron retention in capturing early molecular changes at doses below those impairing viability or gene expression. However, further evaluation using *in vitro* to *in vivo* extrapolation (IVIVE) is needed to determine if intron retention provides PODs that better reflect *in vivo* dose-response compared to gene expression alone. Nonetheless, our results demonstrate the value of intron retention profiling for interpreting chemical MOA through the unique pathways and dose-dependencies revealed. The non-overlapping sets of BMC pathways from gene expression vs. splicing analyses substantiate intron retention as a complementary indicator of biological impacts at lower concentrations.

Future work focusing on the validation and functional characterization of individual alternative splicing events is required to identify individual markers for oxidative stress or other toxicology applications. Follow-up work involving ROS assays and DNA damage assays like COMET to directly quantify oxidative stress upon chemical treatment will be paramount. It will be important to correlate altered splice isoform levels with these functional readouts to determine which splicing events are most associated with oxidative stress and toxicity. Knockdown or overexpression studies focused on shifting isoform ratios will provide further evidence linking alternative splicing to oxidative stress. In addition, studies characterizing the involvement of other splicing events, such as exon skipping, will be important to identify novel biomarkers.

Further visualization of the pathways indicated several processes that were specific to individual chemical-cell line combinations or shared by many chemicals (Supplementary Figure S1). For instance, A549 cells treated with DNCB showed several pathways related to oxygen transport. On the other hand, “Transcriptional Regulation by TP53” and “D-loop structure” pathways were commonly shared with many compounds. Previous research has highlighted the p53 pathways as a marker for oxidative stress (Liu et al., 2020). Similarly, D-loops structures, due to their relaxed structures, have been shown to be more prone to oxidative damage than other mitochondrial DNA regions (Rothfuss et al., 2010).

Taken together, these results demonstrate that splicing analysis may provide another complementary biomarker to standard expression profiling. The non-overlapping pathway sets highlight retained introns as a complementary indicator of biological impacts induced by chemical exposure. Overall, coupling intron retention modeling with gene expression analysis provides a systems perspective.

## Data Availability

The raw sequencing and processed data in this study have been submitted to Gene Expression Omnibus (GEO) with the accession number GSE241672 and can be accessed via this link: https://www.ncbi.nlm.nih.gov/geo/query/acc.cgi?acc=GSE241672.
