# Supplementary figures and images for: Integrating gene expression and splicing dynamics across dose-response oxidative modulators
Source: Front Genet. 2024 May 22;15:1389095. doi: 10.3389/fgene.2024.1389095 (PMC11155298; doi:10.3389/fgene.2024.1389095)

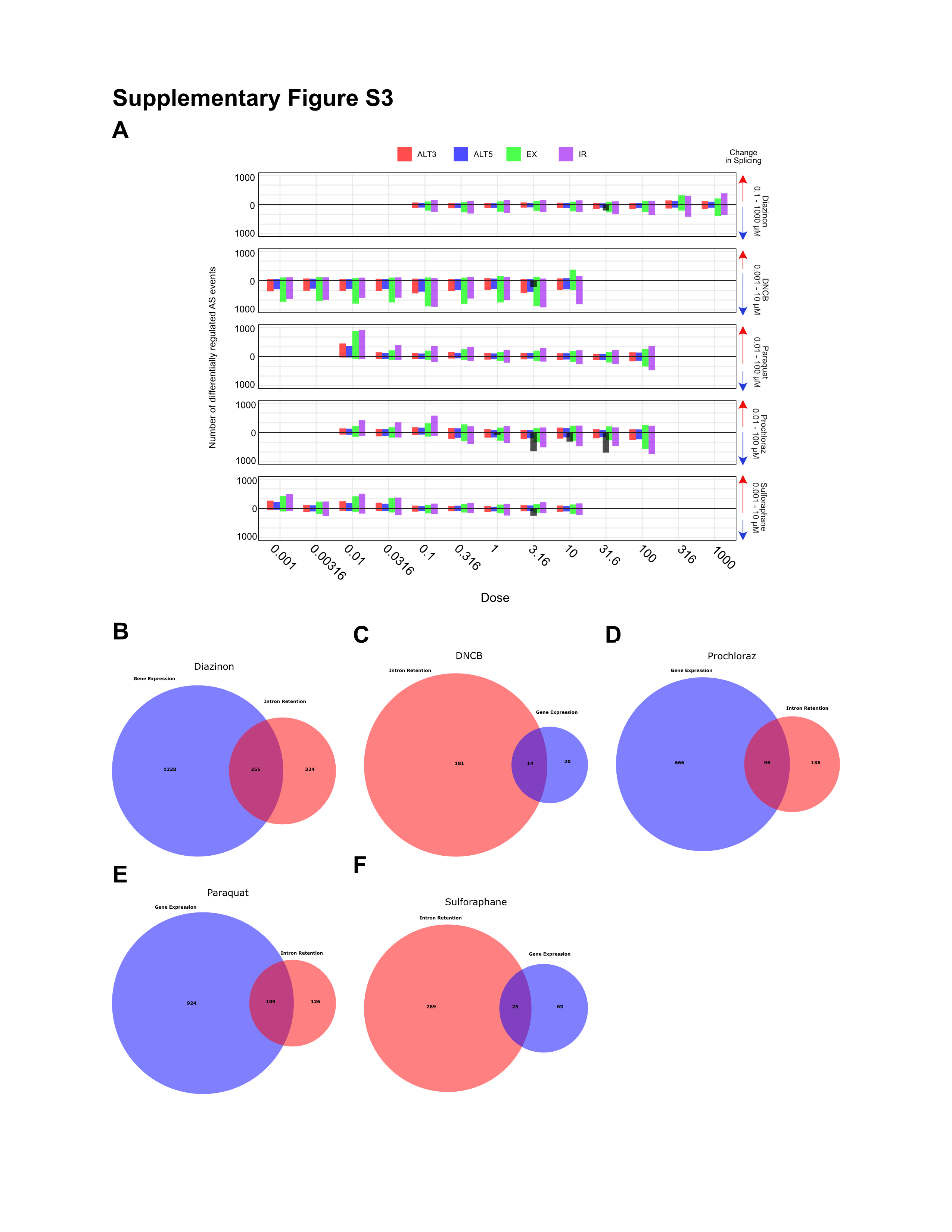

Supplement: Supplementary file 1 [file Image3.TIFF]

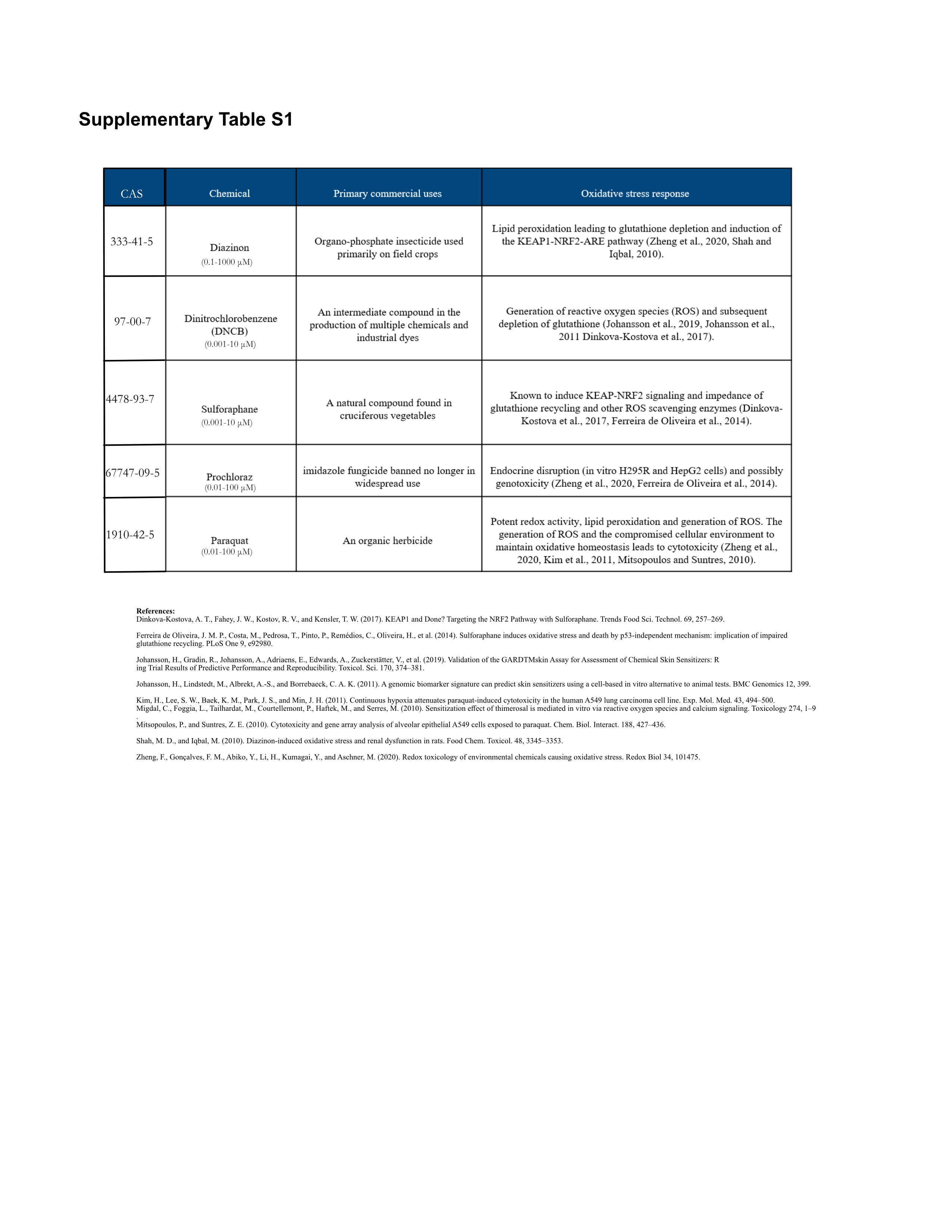

Supplement: Supplementary file 2 [file Image5.TIFF]

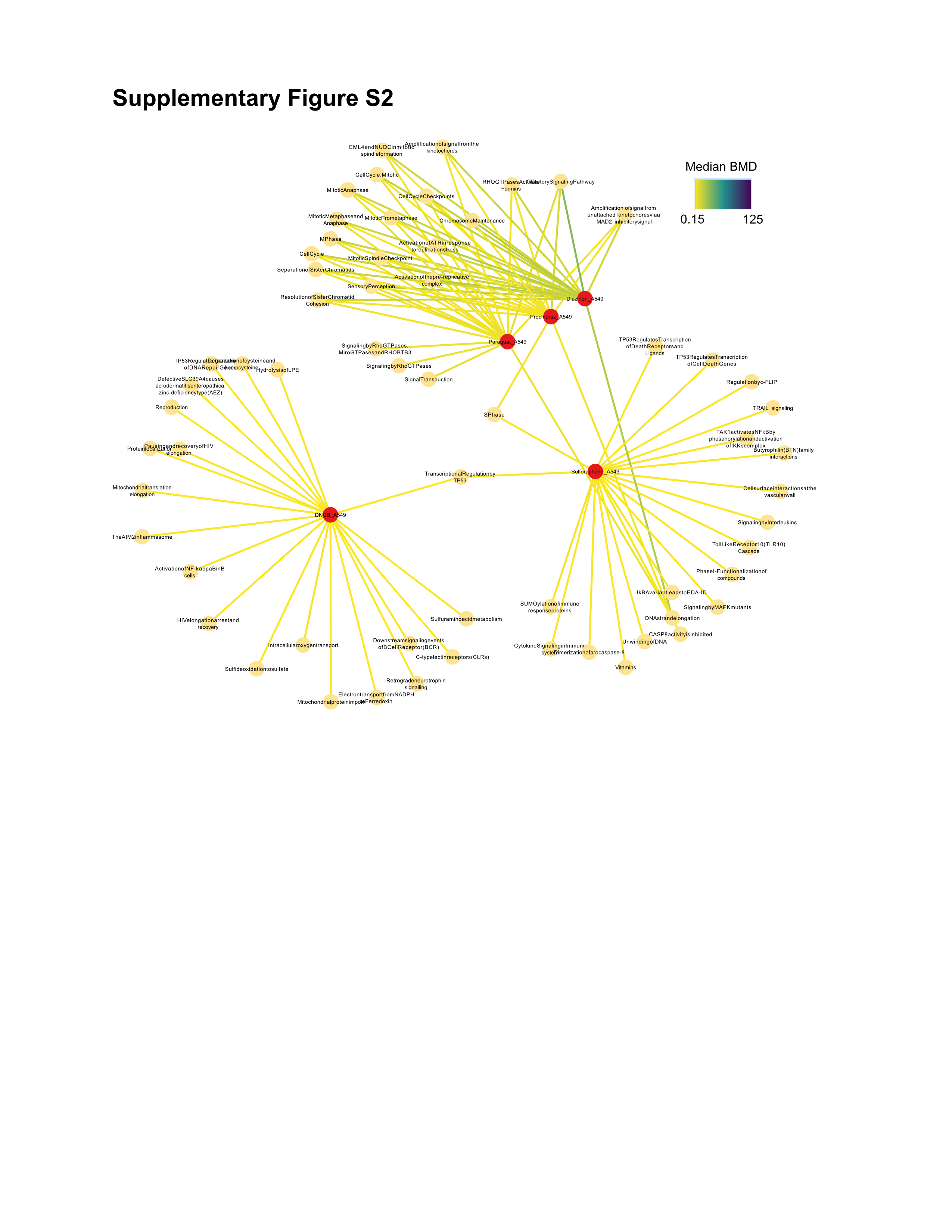

Supplement: Supplementary file 3 [file Image2.PNG]

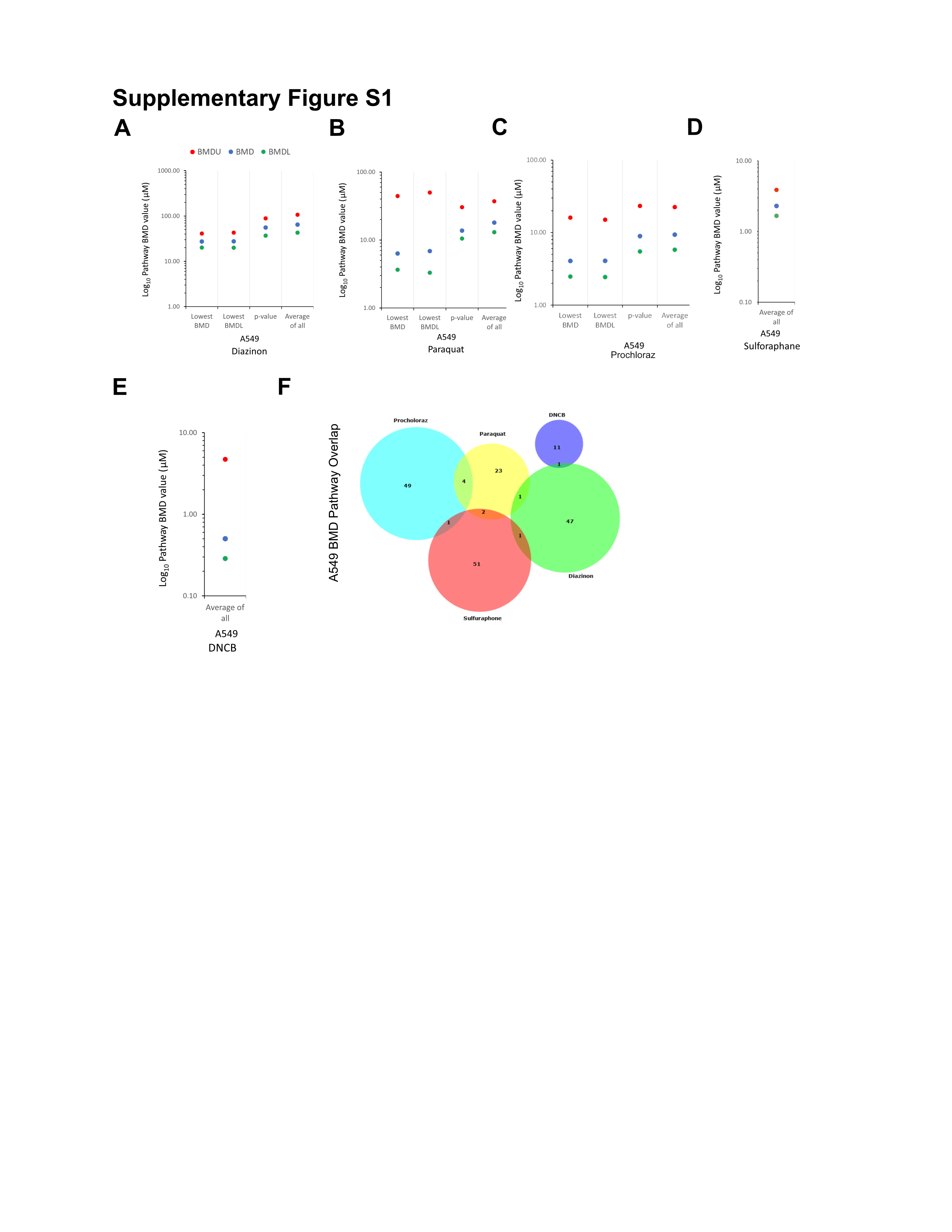

Supplement: Supplementary file 5 [file Image1.PNG]

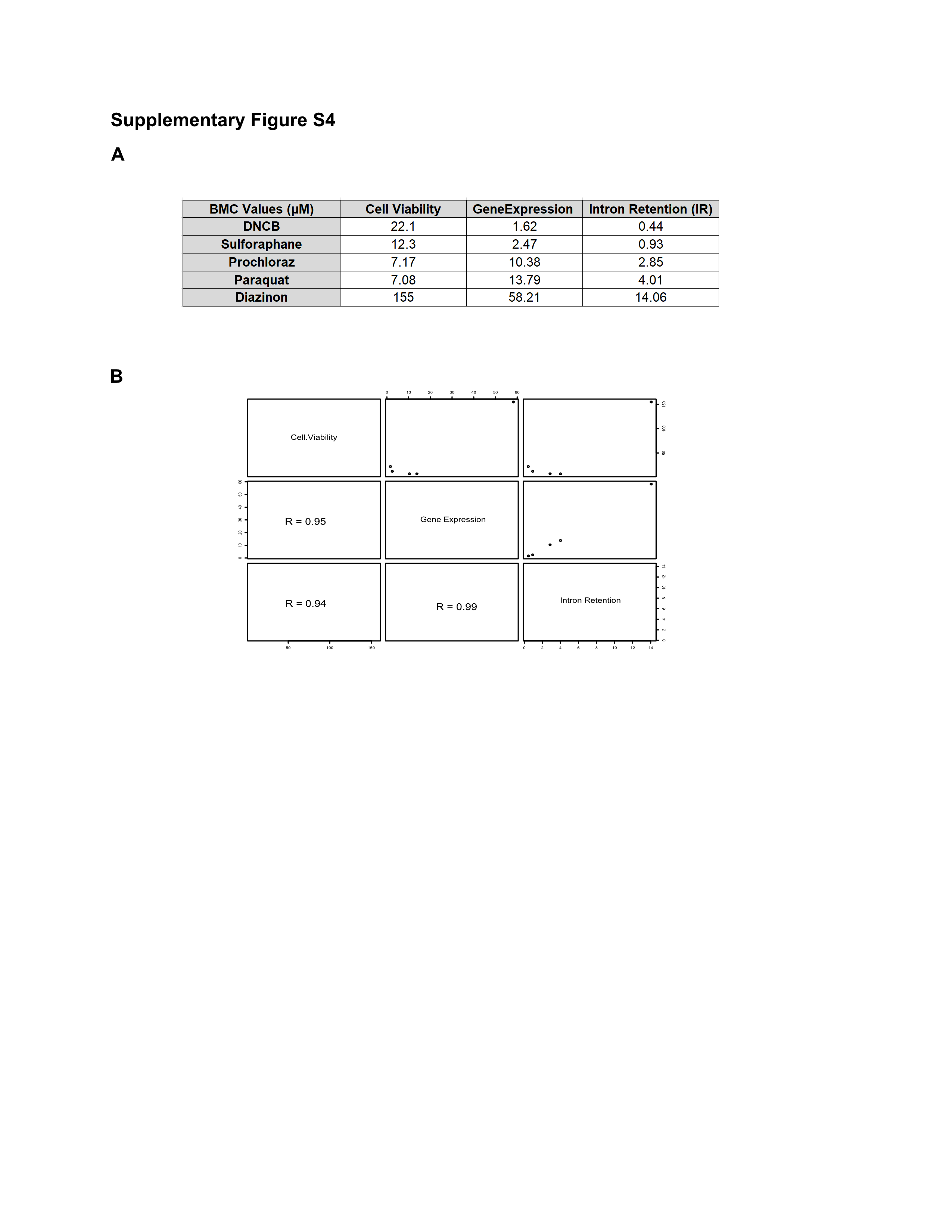

Supplement: Supplementary file 6 [file Image4.TIFF]
